# Simulating stimulus- and TMS-induced interference in short-term memory using a model of prefrontal cortex

**DOI:** 10.1186/1471-2202-15-S1-P141

**Published:** 2014-07-21

**Authors:** Tyler D Bancroft, William E Hockley, Philip Servos, Jeremy Hogeveen

**Affiliations:** 1 Department of Psychology, Wilfrid Laurier University, Waterloo, Ontario, Canada, N2L 3C5

## 

Scalar short-term memory (STM) tasks are those in which the to-be-remembered property of a stimulus can be represented as a scalar quantity – for example, the frequency of a tactile vibration or an auditory pure tone, or the duration or amplitude of a stimulus. Scalar STM tasks have been studied extensively using single-cell methods [1-3], and have proven to be useful model systems for examining behavioural aspects of short-term memory [4-6] and developing computational models [7-10]. In two studies [11,12], we applied a model of prefrontal cortex [9] to experimental datasets [5,13].

In Study 1, we simulated the effects of presenting an irrelevant (distractor) stimulus to experimental subjects during the maintenance period of a vibrotactile scalar STM task by assuming the stimulus was encoded into memory, intruding into the PFC memory store. We were able to replicate previous experimental results [5], and our results also suggested that distractors were only encoded into memory on approximately 50% of trials, consistent with experimental indications that activity in sensory cortex may be inhibited during memory maintenance in order to protect the contents of memory against interference [6,14].

In Study 2, we simulated a previous vibrotactile scalar STM study in which TMS was applied to somatosensory cortex during the maintenance period of the memory task, resulting in decreased performance [13]. We were able to replicate experimental results by assuming that TMS produced increased, noisy neural activity in sensory cortex, which then degraded the contents of the PFC memory store through feedforward interference.
